# Automatic liver tumor segmentation in CT with fully convolutional neural networks and object-based postprocessing

**DOI:** 10.1038/s41598-018-33860-7

**Published:** 2018-10-19

**Authors:** Grzegorz Chlebus, Andrea Schenk, Jan Hendrik Moltz, Bram van Ginneken, Horst Karl Hahn, Hans Meine

**Affiliations:** 1 Fraunhofer Institute for Medical Image Computing MEVIS, Bremen, Germany; 2 Diagnostic Image Analysis Group, Department of Radiology and Nuclear Medicine, Radboud University Medical Center, Nijmegen, The Netherlands; 3 Jacobs University, Bremen, Germany; 4 University of Bremen, Medical Image Computing Group, Bremen, Germany

## Abstract

Automatic liver tumor segmentation would have a big impact on liver therapy planning procedures and follow-up assessment, thanks to standardization and incorporation of full volumetric information. In this work, we develop a fully automatic method for liver tumor segmentation in CT images based on a 2D fully convolutional neural network with an object-based postprocessing step. We describe our experiments on the LiTS challenge training data set and evaluate segmentation and detection performance. Our proposed design cascading two models working on voxel- and object-level allowed for a significant reduction of false positive findings by 85% when compared with the raw neural network output. In comparison with the human performance, our approach achieves a similar segmentation quality for detected tumors (mean Dice 0.69 vs. 0.72), but is inferior in the detection performance (recall 63% vs. 92%). Finally, we describe how we participated in the LiTS challenge and achieved state-of-the-art performance.

## Introduction

According to the World Health Organization, liver cancer was the second most common cause of cancer-induced deaths in 2015. Hepatocellular carcinoma (HCC) is the most common type of primary liver cancer which is the sixth most prevalent cancer^1^. In addition, the liver is also a common site for secondary tumors. Liver therapy planning procedures would profit from an accurate and fast lesion segmentation that allows for subsequent determination of volume- and texture-based information. Moreover, having a standardized and automatic segmentation method would facilitate a more reliable therapy response classification^2^.

Liver tumors show a high variability in their shape, appearance and localization. They can be either hypodense (appearing darker than the surrounding healthy liver parenchyma) or hyperdense (appearing brighter), and can additionally have a rim due to the contrast agent accumulation, calcification or necrosis^3^. The individual appearance depends on lesion type, state, imaging (equipment, settings, contrast method and timing), and can vary substantially from patient to patient. This high variability makes liver lesion segmentation a challenging task in practice.

The problem of liver tumor segmentation has received a great interest in the medical image computing community. In 2008, the MICCAI 3D Liver Tumor Segmentation Challenge^4^ was organized where both manual and automatic methods were accepted. Among the automatic ones, the best method applied an ensemble segmentation algorithm using AdaBoost^5^. Other submitted methods employed adaptive thresholding, region growing or level set methods^6–9^. In more recent years, methods using Grassmannian manifolds^10^ and shape parameterization^11^ were proposed.

Given the variability of liver lesions, a manual design of powerful features is not trivial. Fully convolutional neural networks (FCNs) gained rapidly growing attention in the computer vision community over the last years, because of their ability to learn features automatically from the data. Christ *et al*.^12^ applied two cascaded U-net models^13^ to the problem of liver and liver tumor segmentation. The approach employed one model solely for the liver segmentation and a separate one for the tumor segmentation within a liver bounding box. The final output was refined using a 3D conditional random field.

More recently, the Liver Tumor Segmentation (LiTS) challenge was organized^14^. All top-scoring automatic methods submitted to the two rounds organized in 2017 used FCNs. Han^15^, the winner of the first round, used two U-net like models with long and short skip connections, where the first model was used only for coarse liver segmentation allowing the second network to focus on the liver region. The second model was trained to segment both liver and tumors in one step. The two models worked in 2.5D, i.e., they received five adjacent slices to segment the middle one, which provided the network with the 3D context information. The best method in the second LiTS round was developed by a group from Lenovo Research, China. Their approach employed two neural network ensembles for the liver and tumor segmentation, respectively. The ensembles consisted of 2D and 2.5D U-net models trained with different hyperparameter settings. Other successful methods proposed to train jointly two networks for liver and tumor segmentation^16^ and to exploit 3D information by training a 3D H-DenseUNet architecture using original image data as well as features coming from a 2D network^17^.

This paper focuses on the tumor segmentation task, which follows a separate liver segmentation step that is briefly sketched in the description of the challenge submission. Our contribution on the tumor segmentation task is twofold. First, we show that cascading of a 2D FCN working on a voxel-level with a model trained using hand-crafted features extracted on an object-level leads to a significant reduction of false positive findings and improves the segmentation quality for detected tumors. We provide a detailed description and evaluation of our method, which achieved state-of-the-art results in the LiTS challenge. Second, we report human performance on a subset of the LiTS training data set to put the segmentation quality of automatic methods into perspective.

## Materials and Methods

### Data

In the following, we ran the experiments using the training dataset from the LiTS challenge containing 131 contrast-enhanced abdominal CT scans coming from 7 clinical institutions. The CT scans come with reference annotations of the liver and tumors done by trained radiologists. The in-plane resolution ranges from 0.5 to 1.0 mm and the slice thickness ranges from 0.7 to 5.0 mm. The dataset contains 908 lesions (63% with the longest axial diameter ≥10 mm).

We divided the cases randomly into 3 non-overlapping groups for training, validation and testing containing 93, 6 and 30 cases, respectively. We removed 2 flawed cases due to missing reference tumor segmentation.

### Neural network

#### Architecture

**Figure 1:**
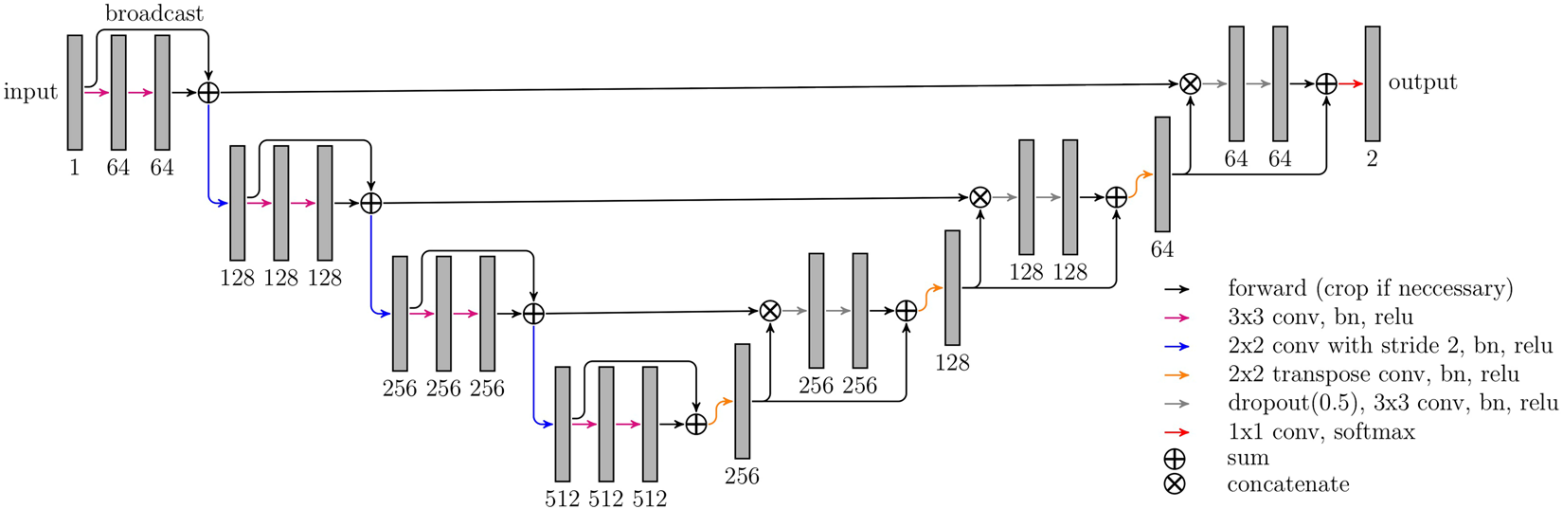
Overview of the neural network architecture. The numbers denote the feature map count.

We employed a U-net^13^ like fully convolutional network architecture (Fig. 1). Our model works on four resolution levels allowing for learning of local and global features. In the contracting (expanding) path convolutions (transposed convolutions) are used to decrease (increase) the spatial resolution and the feature map count is doubled (halved) with each transition. The network contains long skip connections passing feature maps from the contracting path to the expanding path allowing to recover fine details which are lost in the spatial downsampling. We also added short skip connections to have well-distributed parameter updates and to speed up the training^18^. Each convolutional layer uses 3 × 3 filter size and is followed by a batch normalization and a ReLU activation function. We used dropout (p = 0.5) before each convolution in the upscaling path to prevent the network from overfitting.

#### Training

We trained the network using whole axial image slices in the original resolution (size 512 × 512 voxels) and their corresponding labels. Since our architecture is fully convolutional^13^, this is mathematically equivalent to training with many overlapping patches of the receptive field size (here, 92 × 92 voxels), but much more efficient. We used the soft dice coefficient as the loss function computed on the pixelwise softmax of the network final feature map^19^. The loss computation is restrained to a LiTS reference liver mask dilated by 10 mm in order to focus the model on the liver region. To deal with the high class imbalance, we ensured that each mini-batch contains patches where both classes (tumor and background) are present. We computed the parameter updates using the Adam optimizer with 5e-5 learning rate. The model was trained for 10 epochs (approx. 50 k iterations, mini-batch size 6). We reflectively padded the input images with 44 pixels on each side, because we used no zero-padding in the convolutions.

#### Output

The output of the neural network was limited to a liver mask in order to remove false positives found outside of the organ. For the LiTS training dataset we used liver masks provided by the challenge organizers in order to avoid the dependency of the tumor segmentation on the liver segmentation quality. For cases, where a liver mask is not given, the tumor segmentation is preceded by a liver segmentation step (see subsection describing the challenge submission).

### Object-based postprocessing

Based on the training data we observed that some neural network outputs corresponded to false positives, which could easily be identified by their shape and location (e.g. liver/gallbladder boundary). Therefore, we added a post-processing step, which employs a model classifying tumor objects (computed as 3D connected components of the FCN output) into true (TP) and false positives (FP). For that, we trained a conventional random forest classifier (RF) with 256 trees using 36 hand-crafted features carrying information about underlying image statistics, tumor shape and its distance to the liver boundary (the full list of features can be found in the supplementary material). Random forests were chosen for this task because they work well with moderate numbers of training samples and varying feature value distributions. This approach does not allow end-to-end training, because we designed the second model to work on higher level entities (tumor objects instead of voxels) and features that are extracted by an image analysis pipeline from the neural network output^20^. We see this as an advantage, as employment of two separate steps for tumor candidate detection and false positive filtering increases the explainability of the whole system. Whether a tumor is TP or FP was determined using the evaluation code described in Sec. Evaluation.

### Expert performance

In order to put the performance of our automatic method into a perspective, we asked a medical-technical radiology assistant (MTRA) with over 10 years of segmentation experience to manually segment tumors in cases used for the algorithm evaluation. This means that we have two reference annotation sets, which we refer to in the following as “MTRA” and “LiTS”.

### Evaluation

#### Detection

We evaluate the detection performance using metrics based on the Free-Response ROC analysis, which is suitable for experiments involving zero or more decisions per image^21^:

- Recall: Ratio of TP detections to the count of positives in the reference.
- FPs/case: Average count of FPs per case.

Additionally, we compute a ratio of detected tumors with the longest axial diameter ≥10 mm to all such lesions in reference (Recall ≥ 10 mm). The threshold value was derived from the RECIST 1.1 guidelines, where it is used to classify tumor lesions into measurable and non-measurable types^22^.

We define a hit as a situation when the overlap (measured with the Dice index) between output and reference is above a threshold *θ*:

$$DICE({M}^{{\rm{o}}{\rm{u}}{\rm{t}}}[{T}^{{\rm{o}}{\rm{u}}{\rm{t}}}],{M}^{{\rm{r}}{\rm{e}}{\rm{f}}}[{T}^{{\rm{r}}{\rm{e}}{\rm{f}}}]) > \theta $$

*M*^out^ and *M*^ref^ denote output and reference label images where each tumor has a unique label, *T*^out^ and *T*^ref^ are sets of output and reference tumor labels corresponding to each other. Notation *M*[*T*] selects tumors with labels *T* from *M*. The parameter *θ* enables a trade-off between high recall (low *θ*) and high Dice for corresponding tumors (high *θ*). We set *θ* = 0.2 in order to require a significant, but not exact overlap.

**Figure 2:**
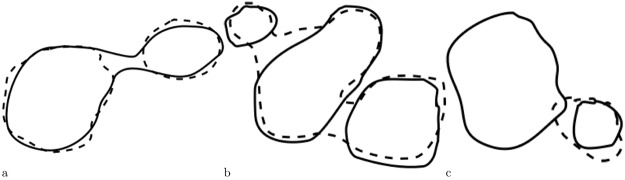
Non-trivial output (*dashed*)/reference (*solid*) correspondences. (**a**) Reference tumor corresponds to two output tumors (**b**) Three reference tumors correspond to one output tumor (**c**) Output tumor corresponds only to the smaller reference tumor.

Determining output/reference tumor correspondence is not trivial, since situations as in Fig. 2 can occur. In Fig. 2a two output tumors ${T}^{{\rm{out}}}=\{{l}_{1}^{{\rm{out}}},{l}_{2}^{{\rm{out}}}\}$ correspond to one reference tumor ${T}^{{\rm{ref}}}=\{{l}_{1}^{{\rm{ref}}}\}$ and if their Dice index > *θ*, then such situation should be counted as one TP. In Fig. 2b one output tumor ${T}^{{\rm{out}}}=\{{l}_{1}^{{\rm{out}}}\}$ corresponds to three reference tumors ${T}^{{\rm{ref}}}=\{{l}_{1}^{{\rm{ref}}},{l}_{2}^{{\rm{ref}}},{l}_{3}^{{\rm{ref}}}\}$ and if their overlap is above *θ*, then such situation counts as three TP.

An algorithm for correspondence establishment of output/reference lesions should aim at maximizing the output/reference overlap. For example, consider Fig. 2c, where the output tumor should correspond only to the smaller reference tumor, since the overlap would decrease if both reference tumors would be considered. To account for *n* : *m* correspondence situations where $n\ne m$, we count merge and split errors for each correspondence. Merge error is defined as $|{T}^{{\rm{ref}}}|-1$, split error as ${\rm{\max }}(0,|{T}^{{\rm{out}}}|-|{T}^{{\rm{ref}}}|)$.

**Algorithm 1:**
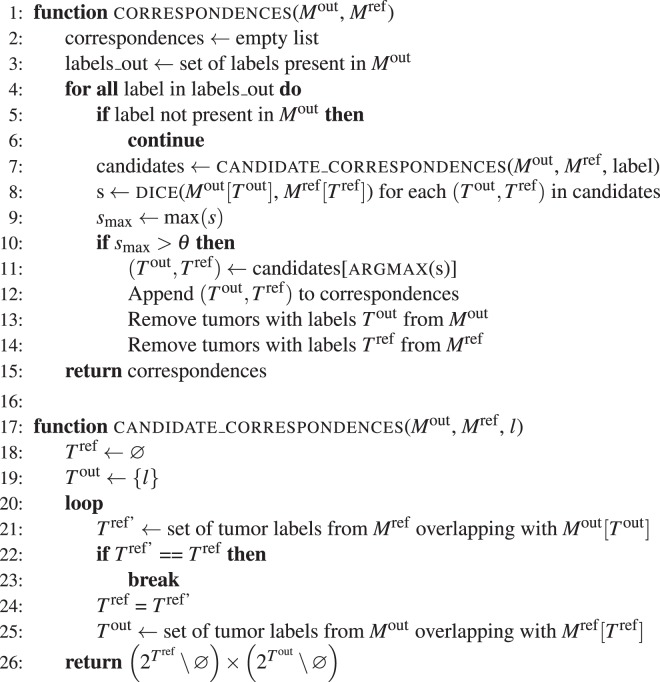
Establishing correspondences between output and reference tumors.

#### Segmentation

The segmentation quality was evaluated using the following measures:

- Dice/case: Computed by taking into account the whole output and reference tumor mask. When both masks are empty, a score of 1 is assigned.
- Dice/correspondence: Computed for each output/reference correspondence.
- Merge error: Sum of per correspondence merge errors.
- Split error: Sum of per correspondence split errors.

Algorithm 1 sketches the code we employed for establishing of correspondences between output and reference tumors.

## Results and Discussion

### Expert performance

**Figure 3:**
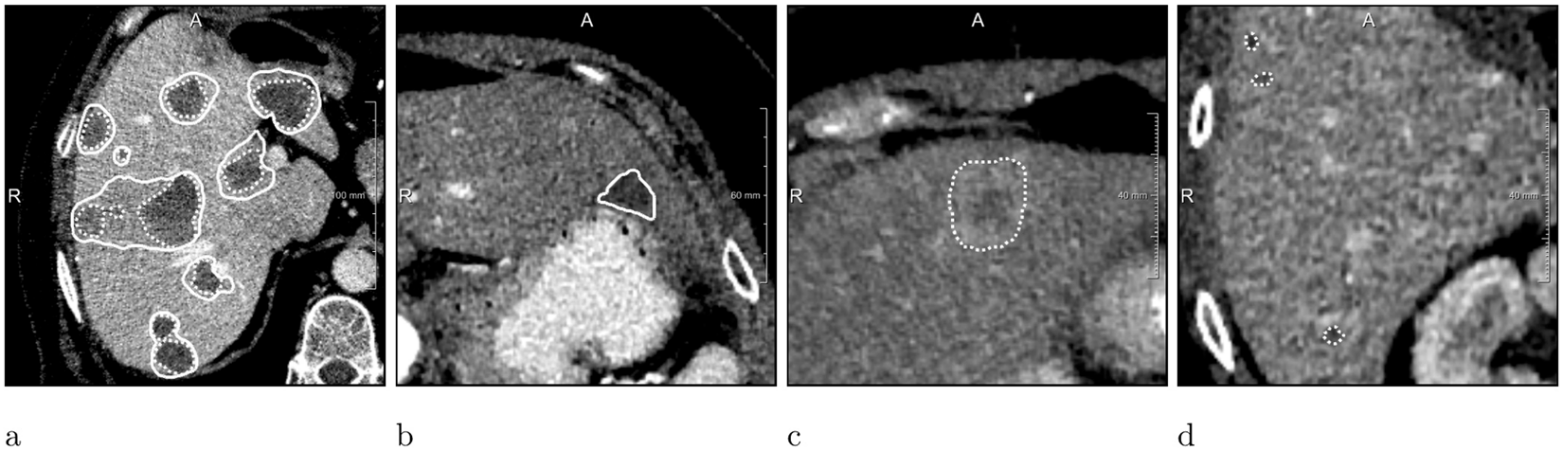
MTRA (*dashed*) vs. LiTS (*solid*) annotations. (**a**) Case with low dice/correspondence (**b**) Case where a LiTS reference tumor was missed (**c**) Case where MTRA found a lesion in a case with no tumors according to LiTS reference (**d**) Case where small additional tumors were found by the MTRA.

The MTRA needed 30–45 min. per case (the segmentation was done without time constraints). The comparison of MTRA with LiTS annotations and vice versa is shown in Table 1. The MTRA missed 11 of the LiTS lesions and found 78 additional ones, which accounts for 0.92 recall and 2.6 FP/case. The LiTS annotations identified correctly only 62% of tumors found by the MTRA. Smaller recall difference was observed for tumors ≥10 mm, meaning that most of the lesions not included by the LiTS reference were small. The segmentation quality was 0.72 dice/correspondence and 0.7 dice/case. Figure 3 shows example cases with major differences between MTRA and LiTS segmentations. There were two cases, where MTRA segmentation got 0 dice/case when compared with the LiTS reference: (i) a tumor was found in a case with no tumors, Fig. 3c, (ii) none of reference tumors were found.

**Table 1:** Mean metric values for human vs. human and computer vs. human comparisons.

	Recall	Recall ≥ 10 mm	FP per case	Dice per case	Dice per correspondence	Merge error	Split error
**Human vs**. **Human**							
MTRA (LiTS)	0.92	0.94	2.6	0.70 ± 0.27	0.72 ± 0.11	11	5
LiTS (MTRA)	0.62	0.85	0.3	0.70 ± 0.27	0.72 ± 0.11	5	12
**Computer vs**. **Human**							
FCN (MTRA)	0.47	0.75	4.7	0.53 ± 0.37	0.72 ± 0.11	7	13
FCN (LiTS)	0.72	0.86	4.6	0.51 ± 0.37	0.65 ± 0.16	12	14
FCN + RF (LiTS)	0.63	0.77	0.7	0.58 ± 0.36	0.69 ± 0.18	11	10

The parentheses denote the dataset used as a reference for the computation of evaluation metrics.

### Neural network

**Figure 4:**
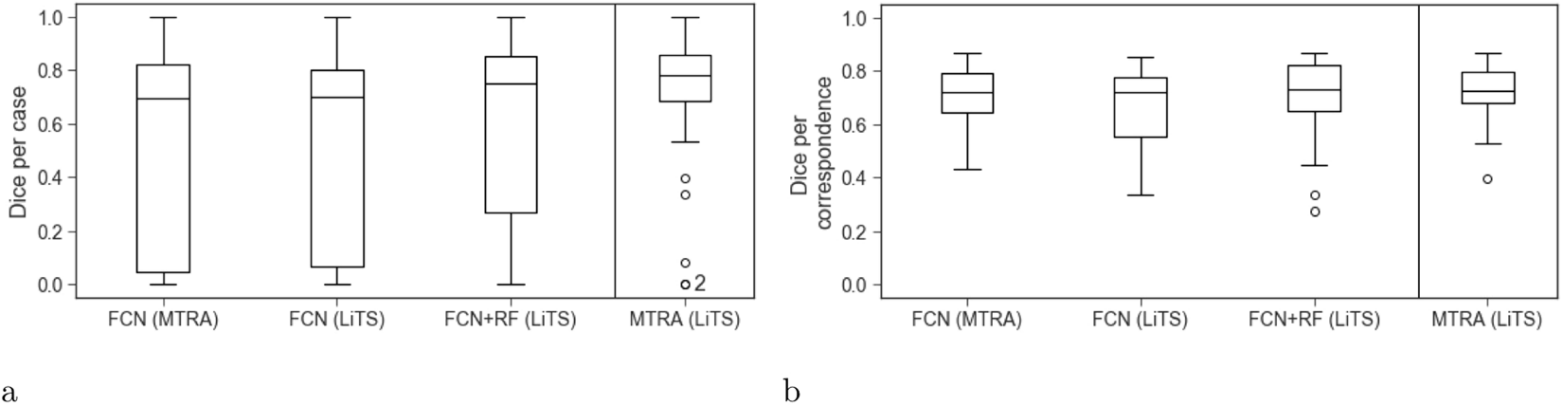
Box plots showing dice per case (**a**) an dice per correspondence (**b**) computed for expert and automatically generated segmentations on 30 test cases.

**Figure 5:**
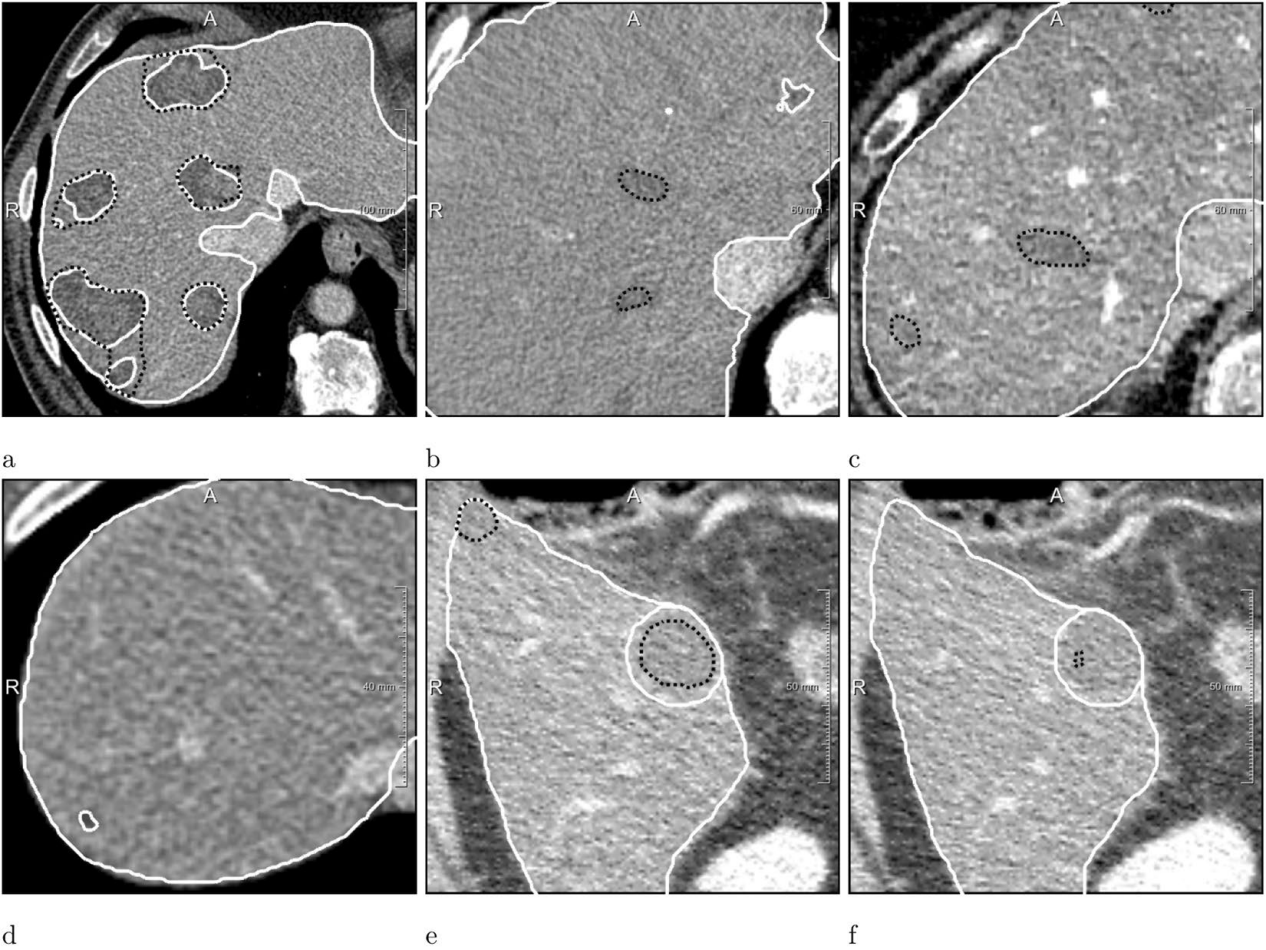
Neural network (*black*) compared with the LiTS (*white*) annotations. (**a**) Case with 0.85 dice/case (**b**,**c**) Cases with 19 and 16 FPs (**d**) Case where a small tumor was not detected (**e**,**f**) Case where tumor segmentation strongly differed on consecutive slices.

The neural network was able to detect 47% and 72% of all tumors present in the MTRA and LiTS annotations, respectively. Tumors with the longest diameter ≥10 mm were detected more reliably than smaller ones. Potentially measurable tumor lesions according to RECIST 1.1 had a recall of 75% and 86%, respectively. The false positive count was similar when comparing with MTRA and LiTS annotations (142 and 138, respectively). The dice/case and dice/correspondence was 0.53 and 0.72 for the MTRA reference and 0.51 and 0.65 for LiTS (see Table 1 and Fig. 4 for details). 7 cases received 0 dice/case score (3 with no reference lesions and 4 where none of small reference lesions was found). Interestingly, the neural network, similar to the MTRA, found a lesion in the case with no tumors in the LiTS reference (Fig. 3c). Figure 5 presents one example of a good segmentation produced by the neural network, as well as examples of different kinds of deviations from the reference.

### Object-based postprocessing

We trained a random forest classifier on features computed for each tumor produced by the neural network from training and validation cases, where only LiTS annotations were available. Therefore, Table 1 reports results only for the LiTS reference. The classifier allowed for a 85% reduction of false positives and had 87% accuracy on test cases: 117 FPs were identified correctly, whereas 13 TPs (9 of which were ≥10 mm) were wrongly rejected. This led to a significant change in FPs, TPs and FNs (all significance tests were done using the Wilcoxon signed-rank test at 0.05 level). The improvement for Dice per correspondence was significant, as opposed to Dice per case, whose increase was achieved by removing all FPs in two cases with no reference tumors. Among five most discriminative features four were shape-based (first eigenvalue, eccentricity, extent along z axis, voxel count). The remaining one described the std. deviation of the distance to the liver boundary (plot showing features sorted according to their importance can be found in the supplementary material).

The main motivation for choosing the random forest classifier was moderate number of training samples. Assuming that a bigger dataset was available, other strategies for object-based post-processing could be investigated. One of possible alternative approaches for false positive reduction would be a multi-view neural network, which learns discriminative features directly from the found tumor candidates^23^.

### Challenge submission and results

Before submission to the LiTS challenge, we trained the neural network further using all cases from the LiTS training dataset. Since the tumor segmentation makes use of liver masks, which were not given for the challenge test cases, we used our own liver segmentation method. For automatic liver segmentation, we trained 3 orthogonal (axial, sagittal, coronal) U-net models with 4 resolution levels on our in-house liver dataset from liver surgery planning containing 179 CTs^24^. We computed segmentations for the 70 challenge test cases ranking third at the MICCAI 2017 LiTS round (leaderboard user name hans.meine). Our submission scored 0.68 and 0.96 dice/case for tumor and liver segmentation, respectively. The tumor dice/case difference between our approach and the best submissions from MICCAI 2017 (IeHealth) and Open leaderboard (xjqi to date) is 0.02 and 0.04, respectively. Our method needs on average 67 s for one case: 43, 16 and 8 s for liver segmentation, tumor segmentation and FP filtering, respectively (Intel Core i7-4770K, 32 GB RAM, GeForce GTX 1080).

## Conclusions

In this work, we described our method for automatic liver tumor segmentation in abdominal CT scans employing a 2D deep neural network with an object-based postprocessing, which ranked third in the second LiTS round at MICCAI 2017. Our tumor segmentation employs a preceding liver segmentation step in order to constrain operation to the liver region and to be able to compute distances from the liver boundary. The object-based analysis step using hand-crafted features allowed for a significant reduction of false positive findings. The fact that the most discriminative features in the postprocessing step were shape-based indicates the importance of 3D information in distinguishing true from false positives. Our method achieves segmentation quality for detected tumors comparable to a human expert and is able to detect 77% of potentially measurable tumor lesions in the LiTS reference according to the RECIST 1.1 guidelines. We observed that the neural network is capable of detecting bigger lesions (the longest axial diameter ≥10 mm) more reliably than smaller ones (<10 mm). We presume, based on the performed comparison of LiTS annotations with those done by an experienced MTRA, that this can be attributed to a bigger inter-observer variability with respect to detection of smaller lesions. We think that the LiTS challenge data collection from multiple sites is a great initiative, that shows not only the variability in imaging, but also some variability in the annotations. This is probably due to the fact that liver tumor segmentation is not part of the daily routine, and that there are no universally agreed on clinical guidelines for this task.

We see the method described in this paper as promising, but it is clear that more work needs to be done to match the human detection performance. Moreover, an evaluation in a clinical setting will be required to assess the clinical utility of automatic liver tumor segmentation methods. Future research directions include evaluation of 3D networks and automation of reporting schemes for the liver.

## Electronic supplementary material

Supplementary Material
