# Supplementary material for: Automatic liver tumor segmentation in CT with fully convolutional neural networks and object-based postprocessing
Source: Sci Rep. 2018 Oct 19;8:15497. doi: 10.1038/s41598-018-33860-7 (PMC6195599; doi:10.1038/s41598-018-33860-7)
Supplement: Supplementary file 1 — Supplementary Material [file 41598_2018_33860_MOESM1_ESM.pdf]

Automatic liver tumor segmentation in CT with fully convolutional neural networks and object-based postprocessing

Grzegorz Chlebus, Andrea Schenk, Jan Hendrik Moltz, Bram van Ginneken, Horst Karl Hahn, and Hans Meine

In the following we present a list of 36 features that were used to train the random forest classifier in the object-based postprocessing stage. The features were extracted for every tumor object, which was computed as a 3D connected component of the neural network output. For feature extraction the output of the neural network, the CT image and the liver distance map were resampled to 1.0 mm isotropic voxel size.

Definitions

T - set of voxel coordinates (x,y,z) belonging to a tumor.
I_T - set of voxel values from the CT image at positions $(x, y, z) \in T$.
D_T - set of voxel values from the liver distance map image at positions $(x, y, z) \in T$.
Tumor moments:

$$m_{ijk} = \sum_{(x,y,z)\in T} x^i y^j z^k$$

Tumor centroid coordinates:

$$(c_x, c_y, c_z) = (\frac{m_{100}}{m_{000}}, \frac{m_{010}}{m_{000}}, \frac{m_{001}}{m_{000}})$$

Tumor central moments:

$$\mu_{ijk} = \sum_{(x,y,z)\in T} (x - c_x)^i (y - c_y)^j (z - c_z)^k$$

Covariance matrix:

$$A = \begin{bmatrix} m_{200} & m_{110} & m_{101} \\ m_{110} & m_{020} & m_{011} \\ m_{101} & m_{011} & m_{002} \end{bmatrix}$$

Feature list

Based on our visual analysis of the tumors found by the FCN for the training data, we decided to include features from three categories: features describing the appearance (i), the location (ii) and the shape (iii) of the tumor object. The features from the first two categories were chosen based on our experience. As features of the third category we used all shape-based features, that our internally used object-based image analysis framework offered. Fig. 1 shows all features sorted according to their importance.

i. Features describing the appearance of the tumor object
1. $\min I_T$
2. $\max I_T$
3. $\sum I_T$
4. average value of I_T
5. standard deviation of I_T
6-7. lower and upper quartile of I_T
8. median of I_T

ii. Features describing the location of the tumor object
9. average value of D_T
10. standard deviation of D_T

iii. Features describing the shape of the tumor object
11. count of tumor voxels
12. count of tumor border voxels
13-15. x, y and z coordinate of the tumor centroid
16-18. tumor extent along x, y and z axis
19-21. eigenvalues of A: λ_1, λ_2, λ_3
22-24. x, y and z coordinate of the first eigenvector of A: (v_x^1, v_y^1, v_z^1)

25. flatness: $1-\sqrt{\lambda_1/\lambda_2}$
26. elongation: $1-\sqrt{\lambda_2/\lambda_3}$
27. eccentricity: $1-27\frac{\det(A)}{(\mathrm{tr}(A))^3}$
28. $\mathrm{atan}(v_x^1/v_y^1)$
29-31. skewness along x, y and z axis: $\mu_{300}/(\sqrt{\mu_{200}})^3$, $\mu_{030}/(\sqrt{\mu_{020}})^3$, $\mu_{003}/(\sqrt{\mu_{002}})^3$
32. ratio between the tumor size and the size of a cube with the same diameter as the diagonal of the tumor bounding box
33. ratio of tumor volume to the volume of a surface area-equivalent sphere
34. ratio of the surface area of a volume-equivalent sphere to the surface area of the tumor
35. ratio of the tumor volume to the volume of a surface area-equivalent ellipsoid
36. ratio of the surface area of a volume-equivalent ellipsoid to the surface area of the tumor

We also tried including local binary patterns (LPB) as texture features, but removed them, because they had no impact on the classifier performance.

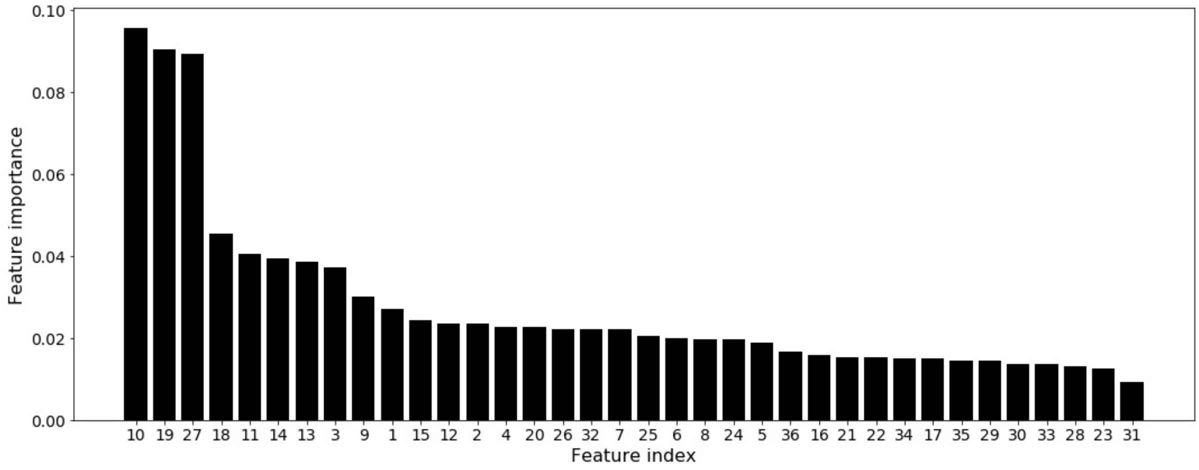

Figure 1: Plot showing features sorted according to their importance.
